# Transthoracic, thoracoabdominal, and transabdominal surgical approaches for gastric cardia adenocarcinomas: a survival evaluation based on a cohort of 7103 patients

**DOI:** 10.1186/s12957-022-02680-5

**Published:** 2022-06-28

**Authors:** Yao Chen, Xue Ke Zhao, Rui Hua Xu, Xin Song, Miao Miao Yang, Fu You Zhou, Ling Ling Lei, Zong Min Fan, Xue Na Han, She Gan Gao, Xian Zeng Wang, Zhi Cai Liu, Ai Li Li, Wen Jun Gao, Jing Feng Hu, Li Guo Zhang, Jin Chang Wei, Fu Lin Jiao, Kan Zhong, Wei Peng Wang, Liu Yu Li, Jia Jia Ji, Xue Min Li, Li Dong Wang

**Affiliations:** 1 State Key Laboratory of Esophageal Cancer Prevention & Treatment and Henan Key Laboratory for Esophageal Cancer Research of The First Affiliated Hospital, Zhengzhou University, Zhengzhou, 450052 Henan Province China; 2 Department of Thoracic Surgery, Anyang Tumor Hospital, Anyang, Henan Province China; 3 Department of Oncology, The First Affiliated Hospital of Henan University of Science and Technology, Luoyang, Henan Province China; 4 Department of Thoracic Surgery, Linzhou People’s Hospital, Linzhou, Henan Province China; 5 Department of Oncology, Tumor Hospital of Linzhou, Linzhou, Henan Province China; 6 Department of Thoracic Surgery, Central Hospital of Xinxiang, Xinxiang, Henan Province China; 7 Department of Pathology and Thoracic Surgery, Linzhou Esophageal Cancer Hospital, Linzhou, Henan Province China; 8 Department of Pathology and Thoracic Surgery, Centre for Health Screening and Endoscopy, Cixian People’s Hospital, Cixian, Hebei Province China

**Keywords:** Surgical approach, Survival, Clinicopathology, Gastric cardia adenocarcinoma (GCA)

## Abstract

**Background:**

This study compared the survival outcomes of different surgical approaches to determine the optimal approach for gastric cardia adenocarcinoma (GCA) and aimed to standardize the surgical treatment guidelines for GCA.

**Methods:**

A total of 7103 patients with GCA were enrolled from our previously established gastric cardia and esophageal carcinoma databases. In our database, when the epicenter of the tumor was at or within 2 cm distally from the esophagogastric junction, the adenocarcinoma was considered to originate from the cardia and was considered a Siewert type 2 cancer. The main criteria for the enrolled patients included treatment with radical surgery, no radio- or chemotherapy before the operation, and detailed clinicopathological information. Follow-up was mainly performed by telephone or through home interviews. According to the medical records, the surgical approaches included transthoracic, thoracoabdominal, and transabdominal approaches. Kaplan–Meier and Cox proportional hazards regression models were applied to correlate the surgical approach with survival in patients with GCA.

**Results:**

There were marked differences in age and tumor stage among the patients who underwent the three surgical approaches (*P* < 0.001). Univariate analysis showed that survival was related to sex, age, tumor stage, and N stage (*P* < 0.001 for all). Cox regression model analysis revealed that thoracoabdominal approach (*P* < 0.001) and transabdominal approach (*P* < 0.001) were significant risk factors for poor survival. GCA patients treated with the transthoracic approach had the best survival (5-year survival rate of 53.7%), and survival varied among the different surgical approaches for different tumor stages.

**Conclusion:**

Thoracoabdominal approach and transabdominal approach were shown to be poor prognostic factors. Patients with (locally advanced) GCA may benefit from the transthoracic approach. Further prospective randomized clinical trials are necessary.

**Supplementary Information:**

The online version contains supplementary material available at 10.1186/s12957-022-02680-5.

## Introduction

Gastric cardia adenocarcinoma (GCA) occurs in the region 1 cm proximal and 2 cm distal to the esophageal-gastric junction. In recent years, the incidence of GCA has risen dramatically in both Western and Eastern countries [1–3]. GCA has a very poor prognosis, with a 5-year survival rate of only approximately 16.7% [4].

To date, the only curative form of therapy for GCA is still surgical resection, and the 5-year survival rate of radical surgery can reach 43–49% [5]. However, the selection of a surgical approach remains controversial and is worthy of further exploration [6–9]. Due to the particularity of the anatomical position of the cardia, general surgeons often adopt the transabdominal approach, while thoracic surgeons are accustomed to using the transthoracic approach. Which surgical approach is more appropriate is a question of interest to many surgeons. However, the current research sample sizes are small, and there is a lack of studies based on large datasets. The aim of this study was to compare the outcomes of different surgical approaches for GCA in a large sample of patients with long-term follow-up to establish an optimal surgical approach for this cancer and provide a reference for the surgical treatment of GCA.

## Materials and methods

### Patients

All patients were from the esophageal and gastric cardia carcinoma clinical diagnosis, pathology, and follow-up databases (1973–2020) established by the State Key Laboratory of Esophageal Cancer Prevention & Treatment and Henan Key Laboratory for Esophageal Cancer Research of The First Affiliated Hospital, Zhengzhou University. We retrospectively analyzed the data of 7103 patients in the database who underwent radical resection and were diagnosed with primary gastric cardia carcinoma after surgery, including 5657 patients treated with the thoracic approach, 942 treated with the thoracoabdominal approach, and 504 treated with the abdominal approach. There were 5600 males and 1503 females with a mean age of 61.4 ± 8.6 years (range: 24–88 years) and 61.5 ± 8.6 years (range: 27–82 years), respectively, and the median age was 62 years. The ratio of men to women was 3.7:1. Written informed consent was provided by each patient. The study protocol was approved by the Medical Ethics Committee of the First Affiliated Hospital of Zhengzhou University.

This retrospective study was based on the following enrollment criteria: (a) patients were diagnosed with GCA by postoperative histopathology, (b) all patients received radical surgery without any radio- or chemotherapy before surgery, and (c) all patients had a clear survival status. Patients who underwent minimally invasive treatment and patients with incomplete surgical approach data were excluded.

### Clinicopathological data collection and standardization

Since 1995, the research team has led more than 50,000 medical students, postgraduates, and volunteers to participate in the construction of an epidemiological survey and database at a high incidence site of esophageal cancer. The clinical diagnosis, treatment, and pathological information were mainly digitalized and standardized under the guidance of experts according to the patient’s hospitalization records and household investigation records. The main digitalized content was the basic information of patients, clinical treatment information, and postoperative pathological information, and the postoperative pathological indices were standardized according to the American Joint Committee on Cancer/Union International Against Cancer (AJCC/UICC) TNM staging guidelines (6th edition, 2002) for esophageal cancer. In our database, when the epicenter of the tumor was at or within 2 cm distally of the esophagogastric junction, the adenocarcinoma was considered to originate from the cardia and was considered a Siewert type II cancer.

### Follow-up

This study mainly used letters (mainly in the 1970s and 1990s), telephone interviews, home call, and visits from village doctors to directly contact the patients or their families or followed up the patients by querying the new cooperative medical database, the medical security bureau database, or the citizen death information registration management system. In the first year after discharge, patients were followed up every 3 months and then once annually until the final event (death) occurred. The last follow-up was completed on January 25, 2021. The diagnosis time of the patient was defined as the date when patients were confirmed to have GCA by histopathology. The survival period was from the time of diagnosis to death (endpoint event) or the time of the last follow-up and was calculated in years.

### Surgical approach

In this study, which was based on the medical records, the surgical approaches mainly included transthoracic, thoracoabdominal, and transabdominal approaches. Notably, due to the small number of Ivor Lewis procedures (51 cases), these patients were incorporated into the transthoracic approach group.

In the transthoracic approach, patients were positioned in the right lateral decubitus position, and thoracotomy was performed at the left sixth or seventh intercostal space. The lower thoracic segment of the esophagus was separated, the thoracic lymph nodes were removed, access to the abdomen was achieved through a diaphragmatic incision, and abdominal lymph node dissection was completed. The esophagus and part of the stomach were cut off at least 5 cm from the tumor in the lower part of the esophagus, and esophagogastrostomy was performed just below or above the aortic arch [10].

In the thoracoabdominal approach, the patients were placed in a right lateral decubitus position at an angle of approximately 90° to allow for simultaneous exposure of the tip of the scapula and the midline of the abdomen. An initial laparotomy was performed to exclude metastatic disease and to ensure tumor resectability. The thoracic incision was then continued through the sixth or seventh intercostal space with excision of a segment of costal cartilage. The diaphragm was incised circumferentially to gain maximal exposure while preserving the left anterior phrenic nerve. The tumor was resected with a clearance of at least 10 cm proximally and 5 cm distally, and reconstruction was performed with a gastric pull-up technique [11].

The transabdominal approach indicated a median laparotomy. First, abdominal lymph node dissection was performed. Then, the peritoneum was cut at the esophageal hiatus, the lower esophagus was freed and pulled downward, the proximal stomach or the whole stomach and the lower esophagus (5–6 cm) were removed, and esophagogastric anastomosis was finally performed; for total gastrectomy, esophageal jejunal anastomosis or Roux-en-Y anastomosis was performed [12].

### Statistical analysis

Statistical analysis was performed using SPSS for Windows 25.0 (IBM Corp., Armonk, NY, USA) Continuous variables are expressed as the mean and were compared using the Kruskal–Wallis test, whereas categorical variables were compared using the chi-square test. The effect of each variable on survival was analyzed by the Kaplan–Meier method, and survival curves were generated using the log-rank test to determine univariate significance. The interaction among multiple variables was analyzed by a Cox proportional hazards regression model to screen for independent prognostic factors. For multiple comparisons, Bonferroni’s correction was used.

## Results

### Patient characteristics

Of the 7103 patients with GCA, 5657 (80%) underwent the transthoracic approach, 942 (13%) underwent the thoracoabdominal approach, and 504 (7%) underwent the transabdominal approach. A comparison of the demographic data and histopathological tumor characteristics showed marked differences among the patients treated with the three surgical approaches. The patients were predominantly male. No significant differences with respect to the distribution of sex (*P* = 0.24) and N stage (*P* = 0.06) were evident among patients treated with different surgical approaches.

Patients under 40 years of age with GCA were more likely to undergo the thoracoabdominal approach than the transthoracic approach. Patients aged 40–49 years underwent the thoracoabdominal approach more often than the transabdominal approach, and patients aged 50–59 years underwent the transthoracic approach more often than the transabdominal approach. For patients aged 60–69 years, resection with the transabdominal approach was performed more often than that with the transthoracic approach. There was no difference in the distribution of surgical approaches for 70- to 79-year-old patients, whereas patients over 80 years old were more likely to be treated by the transabdominal approach.

Most patients were had intermediate stage (1 + 3) disease (up to 92%). Patients with stage 1 disease more commonly underwent the transabdominal approach, and patients with stage 2 disease more commonly underwent the transthoracic approach. There was no difference in the distribution of surgical approaches among patients with stages 0 and 3 disease, and those with advanced stage (IV) disease were least inclined to be treated by the transthoracic approach (Table 1).

**Table 1:** The distribution of clinicopathological characteristics in 7103 GCA patients treated with different surgical approaches, *n* (%)

Characteristics	No. of patients examined	Transthoracic (*n* = 5657)		Thoracoabdominal (*n* = 942)		Transabdominal (*n* = 504)		*p-*Value
Sex								0.24
Male	5600	4439	(79)	762	(81)	399	(79)	
Female	1503	1218	(21)	180	(19)	105	(21)	
Age (years)								< 0.001*
< 40	64	44	(1)	15	(2)	5	(1)	
40–49	550	430	(7)	92	(10)	28	(5)	
50–59	2061	1692	(30)	258	(27)	111	(22)	
60–69	3145	2487	(44)	408	(43)	250	(50)	
70–79	1224	966	(17)	161	(17)	97	(19)	
≥ 80	59	38	(1)	8	(1)	13	(3)	
Tumor stage								< 0.001†
0	30	24	(1)	4	(1)	2	(1)	
I	328	250	(4)	38	(4)	40	(8)	
II	2991	2448	(43)	397	(42)	146	(29)	
III	3541	2813	(50)	454	(48)	274	(54)	
IV	213	122	(2)	49	(5)	42	(8)	
N stage								0.06
N0	2737	2218	(39)	344	(36)	175	(35)	
N1	4366	3439	(61)	598	(64)	329	(65)	

*Significant difference between the transthoracic and thoracoabdominal groups in the < 40 subgroup; significant difference between the thoracoabdominal and transabdominal groups in the 40 subgroups; significant difference between the transthoracic and transabdominal groups in the 50 subgroups and 60 subgroups; no significant difference among all three groups in the 70 subgroups; significant difference between the transabdominal and transthoracic and between the transabdominal and thoracoabdominal groups in the 80 subgroups

†No significant difference among all three groups among patients with stages 0 and 3 disease; significant difference between the transabdominal and transthoracic and between the transabdominal and thoracoabdominal groups among patients with stages 1 and 2 disease; significant difference between the transthoracic and thoracoabdominal and between the transthoracic and transabdominal groups among patients with stage 4 disease

### Surgical outcome

**Fig. 1:**
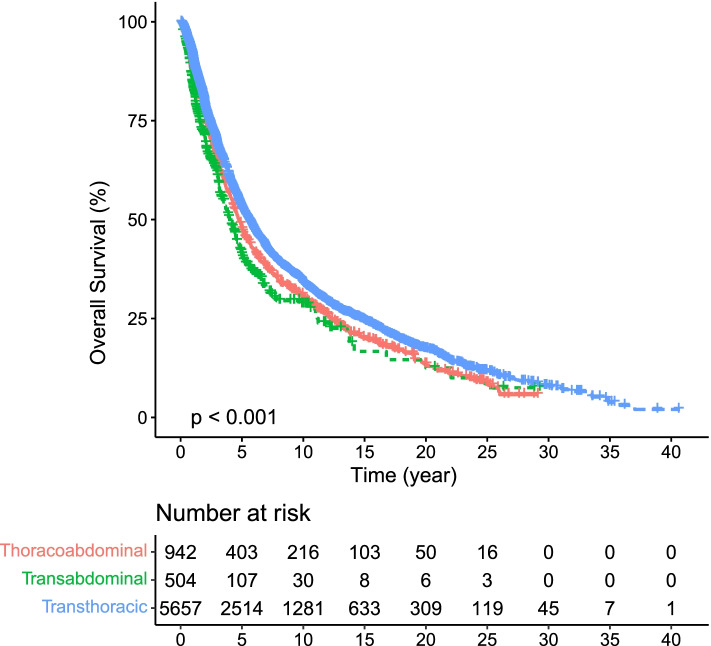
Kaplan–Meier curves comparing different surgical approaches in 7103 GCA patients

The survival analysis of different surgical approaches in GCA patients showed that the transthoracic approach had the highest survival rate (5-year survival rate of 53.7%), while the 5-year survival rate of the thoracoabdominal approach was similar to that of the transabdominal approach (47.9% vs. 40.9%, *P* = 0.04) (Fig. 1).

To exclude the influence of disease stage on survival rate, all cases were stratified according to tumor stage (there were too few patients with stage 0 disease, and these patients were incorporated included with the stage 1 group for analysis). The results showed that when patients had stage 0/1 (*P* = 0.60) or 2 (*P* = 0.41) disease, there was no significant difference in survival rate among different surgical approaches (Supplemental Fig. S1 A–B). When patients had stage 3 disease, the survival rate of patients who underwent the transthoracic approach was significantly different from that of patients who underwent the other two approaches (transthoracic vs. thoracoabdominal, *P* = 0.002; transthoracic vs. transabdominal, *P* < 0.001); the survival rate of patients who underwent the transthoracic approach was the highest (5-year survival rate of 43.9%), while the thoracoabdominal approach offered no survival benefit over the transabdominal approach in these patients (*P* = 0.06) (Supplemental Fig. S1C). When patients had stage 4 disease, there was no significant difference in the 5-year survival rate among different surgical approaches (transthoracic vs. thoracoabdominal, *P* = 0.02; transthoracic vs. transabdominal, *P* = 0.07; thoracoabdominal vs. transabdominal, *P* = 0.87) (Supplemental Fig. S1D).

Similarly, when GCA patients had N0 stage disease, there was no significant difference in survival among the different surgical approaches (*P* = 0.31) (Supplemental Fig. S2A). While the patients had N1 stage disease, the transthoracic approach was best in terms of survival (transthoracic vs. thoracoabdominal, *P* < 0.001; transthoracic vs. transabdominal, *P* < 0.001), and the thoracoabdominal approach offered no survival benefit over the transabdominal approach in these patients (*P* = 0.06) (Supplemental Fig. S2B).

### Survival and prognostic factors

Univariate analysis showed that sex, age, tumor stage, and N stage were related to survival (*P* < 0.001). The median survival time with the transthoracic approach (5.66 y) was longer than that with the thoracoabdominal (5.66 y vs. 4.66 y, *P* < 0.001) and transabdominal (5.66 y vs. 3.84 y, *P* < 0.001) approaches in males (Table 2).

**Table 2:** Univariate analysis of the effects of clinicopathological characteristics on survival in 7103 GCA patients treated with different surgical approaches

Characteristics	Median survival (95% *CI*)			*P*^a^	*P*^b^
	**Transthoracic**	**Thoracoabdominal**	**Transabdominal**		
Sex					
Male	5.66 (5.37–5.94)	4.66 (4.27–5.05)	3.84 (3.15–4.53)	< 0.001*	< 0.001
Female	5.69 (5.08–6.29)	5.73 (3.53–7.94)	4.79 (3.60–5.98)	0.32	
Age (years)					
< 40	5.02 (1.93–8.12)	3.29 (0.34–6.24)	Not reached	0.34	< 0.001
40–49	7.27 (5.23–9.31)	7.27 (5.33–9.22)	3.55 (1.98–5.12)	0.10	
50–59	7.04 (6.34–7.74)	4.95 (4.19–5.71)	6.268 (4.22–8.32)	0.02	
60–69	5.68 (5.31–6.04)	4.73 (4.05–5.40)	4.088 (3.33–4.84)	0.002†	
70–79	4.00 (3.65–4.36)	3.50 (2.19–4.82)	2.70 (1.72–3.68)	0.005‡	
≥ 80	3.51 (2.84–4.18)	3.55 (1.49–5.61)	1.22 (0.56–1.88)	0.16	
Tumor stage					
0/I	13.09 (8.90–17.27)	9.39 (4.49–14.29)	Not reached	0.60	< 0.001
II	7.70 (7.08–8.33)	7.02 (5.26–8.78)	6.95 (2.61–11.28)	0.41	
III	4.23 (4.02–4.45)	3.79 (3.31–4.27)	3.16 (2.58–3.73)	< 0.001	
IV	4.99 (3.60–6.38)	3.09 (1.51–4.68)	3.01 (1.53–4.50)	0.03	
N stage					
N0	8.99 (8.23–9.75)	8.51 (6.38–10.64)	6.95 (2.67–11.22)	0.31	< 0.001
N1	4.46 (4.26–4.67)	3.93 (3.55–4.31)	3.16 (2.66–3.67)	< 0.001	

*Transthoracic vs. thoracoabdominal, *P* < 0.001; transthoracic vs. transabdominal, *P* < 0.001; thoracoabdominal vs. transabdominal, *P* = 0.08

†Transthoracic vs. thoracoabdominal, *P* = 0.12; transthoracic vs. transabdominal, *P* = 0.001; thoracoabdominal vs. transabdominal, *P* = 0.08

‡Transthoracic vs. thoracoabdominal, *P* = 0.20; transthoracic vs. transabdominal, *P* = 0.001; thoracoabdominal vs. transabdominal, *P* = 0.14

Patients aged 60 to 79 with GCA who were treated by the transthoracic approach had a significantly longer survival time than those treated with the transabdominal approach (*P* = 0.001). However, when the patients were younger than 50 years or older than 80 years, there was no significant difference in survival among the different surgical approaches (*P* > 0.05) (Table 2).

A Cox proportional hazards model was used to analyze the influence of sex, age, tumor stage, and surgical approach on the prognosis of patients with GCA. The parameters used were as follows: regression α in = 0.05, α out = 0.1, and forward LR method; all the above variables were entered into the regression model. As shown in Table 3, thoracoabdominal and transabdominal approach were significant risk factors for poor survival. Comparing the three surgical approaches, the transthoracic approach had the lowest risk of death, followed by the thoracoabdominal approach (*HR* = 1.16, 95.0% *CI*: 1.07–1.26), and the transabdominal approach had the highest risk of death (*HR* = 1.31, 95.0% *CI*: 1.16–1.49).

**Table 3:** Univariable analysis and Cox multivariate regression analysis for 7103 GCA patients

Variable	Univariable analysis		Multivariable analysis	
	***HR* (95% *CI*)**	***p-*Value**	***HR* (95% *CI*)**	***p-*Value**
Sex				
Female vs. male	0.90 (0.84–0.97)	0.004	0.88 (0.82–0.95)	< 0.001
Age (years)				
40–49 vs. < 40	0.79 (0.57–1.08)	0.14	0.89 (0.65–1.22)	0.46
50–59 vs. < 40	0.93 (0.69–1.26)	0.63	1.05 (0.77–1.42)	0.76
60–69 vs. < 40	1.15 (0.85–1.56)	0.36	1.31 (0.97–1.77)	0.08
70–79 vs. < 40	1.64 (1.20–2.22)	0.002	1.87 (1.37–2.53)	< 0.001
≥ 80 vs. < 40	2.49 (1.65–3.75)	< 0.001	2.59 (1.72–3.91)	< 0.001
Tumor stage				
II vs. 0/I	1.54 (1.27–1.85)	< 0.001	1.56 (1.29–1.88)	< 0.001
III vs. 0/I	2.54 (2.11–3.07)	< 0.001	2.60 (2.16–3.13)	< 0.001
IV vs. 0/I	2.37 (1.87–3.01)	< 0.001	2.36 (1.86–3.00)	< 0.001
Surgical approach				
Thoracoabdominal vs. transthoracic	1.15 (1.06–1.25)	0.001	1.16 (1.07–1.26)	< 0.001
Transabdominal vs. transthoracic	1.38 (1.21–1.56)	< 0.001	1.31 (1.16–1.49)	< 0.001

*95% CI* 95% confidence interval, *HR* hazard ratio

### Surgical period analysis

We divided all patients into 3 groups according to diagnosis date (1974–1999, 2000–2011, and 2012–2020). The survival rates were similar among the different surgical approaches in 1974–1999 (*P* = 0.31) (Supplementary Fig. S3A). In 2000–2011, the survival rate of the transthoracic approach was significantly different from that of the other two approaches (transthoracic vs. thoracoabdominal, *P* < 0.001; transthoracic vs. transabdominal, *P* = 0.002), and the transthoracic approach had the best survival; however, no significant difference was found between the thoracoabdominal and transabdominal approaches (*P* = 0.60) (Supplemental Fig. S3B). In 2012–2020, the survival rates of the transthoracic and transabdominal approaches were better than those of the thoracoabdominal approach (transthoracic vs. thoracoabdominal, *P* < 0.001; thoracoabdominal vs. transabdominal, *P* = 0.01), but there was no significant difference between the transthoracic and transabdominal approaches (*P* = 0.37) (Supplemental Fig. S3C).

### Lymph node dissection conditions

A comparison of the number of positive lymph nodes (Supplemental Table S1, *P* = 0.78) using the Kruskal–Wallis test did not show a significant difference among the three surgical approaches. However, the transabdominal approach allowed for significantly more lymph nodes to be removed (Supplemental Table S1, *P* < 0.001) than the other two approaches.

## Discussion

Surgical resection is undoubtedly the most important treatment for GCA. The special anatomic position of the cardia and the uniqueness of lymphatic reflux contribute to the increased difficulty of surgical resection. Tumor removal, complete cleaning of the lymph nodes, and selection of a reasonable surgical approach can improve the level of radical treatment for GCA. There are three traditional surgical approaches: transabdominal, thoracoabdominal, and transthoracic. However, there is no uniform standard approach for GCA resection; which approach is most beneficial has been a hot topic of interest to surgeons.

To the best of our knowledge, this is the largest study of GCA patients in terms of clinicopathological features and treatment outcomes. In our study sample, the male sex predominated. Compared with male sex, female sex was a protective factor for the prognosis of GCA (*HR* = 0.88, 95.0% *CI*: 0.82–0.95). This was consistent with multiple reports [13–15]. In addition to genetic susceptibility, this difference may be related to the higher proportion of males with unhealthy lifestyle habits, such as smoking and drinking, [16] but the underlying mechanism needs further study. In the present study, it was noteworthy that 0.8% of GCA patients over 80 years of age underwent surgery. As the average life expectancy increases, the treatment of elderly patients with GCA has attracted increasing attention. As long as their cardiopulmonary function allows, elderly patients may maintain a positive attitude regarding treatment [17, 18]. By comparing the clinicopathological characteristics of the transthoracic group, the thoracoabdominal group, and the transabdominal group, it was found that there were some differences among the three groups. These differences may be because this study is a retrospective study and is influenced by human factors (the choices of the patients and doctors), but these differences are also related to GCA at the junction of the esophagus and the stomach. It is noteworthy that most of the patients underwent the transthoracic approach, which may be due to the following reasons: (1) in this study, more than 80% of the patients were from the highest incidence areas for GCA in Henan province in northern China, where physicians prefer the transthoracic approach. To some extent, this leads to poor representation in the cohort. Further research should include hospitals in high- and low-incidence areas so that the database can provide an overview of surgical treatment for GCA in all of China. (2) In the past few decades, GCA has been classified as a thoracic disease in China, so most patients underwent a transthoracic approach. Overall, this may be due to differences in the surgeons’ training, their professional habits and individual preferences, and the patients’ conditions. However, although not comprehensive, this study shows real-world data.

There is no doubt that each surgical approach has its advantages and disadvantages, and the most important criterion is survival rate after surgery [19]. Survival, as the most important index to evaluate the effect of various clinical treatments, was the main observed outcome in this study. Using a large dataset, we showed that survival was best through the thoracic approach. Recent reports on the comparisons between different surgical approaches for GCA show that many debates still exist. Several reports [20, 21] have found that the transabdominal approach has the advantages of minimal trauma, minimal blood loss, short operation time, significantly reduced interference to the respiratory system and circulation, and easy recovery. Therefore, it is especially suitable for frail elderly patients with poor cardiopulmonary function. This may be the reason why elderly patients over 80 years of age in our study preferred the transabdominal approach. However, the transabdominal approach cannot reach the inferior paraesophageal region, and the lymph node metastasis rate in this site is 18.2% [22]. When the tumor invades the lower esophagus, the resection is often incomplete, which easily leads to residual upper esophageal cancer. Some studies [23, 24] believe that the thoracoabdominal approach enables a good visual field, which is convenient for total gastrectomy and thorough lymph node dissection and facilitates the combined resection of multiple organs. Furthermore, some researchers [6, 8, 25] have proposed that the thoracoabdominal approach is very traumatic, time-consuming, and labor-intensive and is associated with many complications and high mortality. Therefore, the researchers concluded that the thoracoabdominal approach cannot be justified to treat Siewert type 2 tumors of GCA. Similar to our results, other investigators [26–28] have recommended the use of the transthoracic approach. The transthoracic approach exposes the lower esophagus and gastric fundus well and is convenient for surgical operations. Surgery through this approach can fully remove the lower esophagus to ensure that there is no residual tumor at the resection margin. Moreover, it is conducive to sweeping the lymph nodes in the thoracic cavity, enables convenient anastomosis, and leads to less trauma than the thoracoabdominal approach. Consequently, this approach has been widely used in clinical practice. However, on the other hand, the transthoracic approach does not easily expand the scope of gastrectomy, and it is not ideal for the removal of abdominal lymph nodes; additionally, compared to the transabdominal approach, it has a greater impact on the patient’s cardiopulmonary function [29]. Compared with China, Western countries are more inclined to use the transhiatal approach. Researchers in Western countries [7, 29, 30] believe that the transhiatal approach has fewer complications, low mortality, and a short hospital stay, but the survival rate is similar to that of the transthoracic approach. Mariette’s review [9] published in Lancet Oncology in 2011 concluded that two resection procedures are possible for Siewert type 2: total gastrectomy with partial esophagectomy via the abdominal approach or superior polar oesogastrectomy via the transthoracic or transhiatal approach.

To better guide decisions in clinical practice, we further stratified all cases according to tumor stage and found that when the tumor was in the early or advanced stage, the surgical approach did not affect survival. However, the transthoracic approach was the best choice for locally advanced disease. Similar to tumor stage, when the tumor was in the N0 stage, the choice of surgical approach did not affect the survival rate. When the tumor was in the N1 stage, the transthoracic approach had the highest survival rate. Therefore, the present study indicated that patients with (locally advanced) GCA may benefit from the transthoracic approach.

Notably, in addition to patient sex, age, and tumor stage, surgical approach was identified as a prognostic factor independently associated with overall survival after surgical resection of GCA in the Cox proportional hazards regression model in this study. The choice of surgical approach had significant impacts on long-term survival. Our results are consistent with those of Blank S et al. [31, 32]. In their study, surgical approach was an independent factor affecting the prognosis of patients with GCA. However, our results are inconsistent with those of other reports [33, 34] that found that surgical approach was not associated with survival. These inconsistencies might be explained by the adjustment range of confounding variables and may also be related to sample size and follow-up quality.

Our study showed that survival varied with different surgical approaches in different periods. From 1974 to 1999, the surgical technique was still immature, so there was no difference in survival among different surgical approaches. With the progression of technology, the transthoracic approach has shown strong advantages. Interestingly, in recent years, the advantages of the transabdominal approach have gradually emerged. Although data regarding lymph node metastasis status at each location were incomplete in this study, we observed that the transabdominal approach allows for significantly more lymph nodes to be removed than the other two approaches; however, this did not completely translate into a survival advantage, which may be related to the method of removing lymph node metastasis. The transabdominal approach mainly removed abdominal lymph nodes and was not conducive to thoracic lymphadenectomy. Meanwhile, we presumed that it was also possibly because there was no significant difference in the number of positive lymph nodes between any two approaches.

In summary, the selection of a reasonable approach should focus on radical treatment of the tumor, ensuring postoperative anatomical and physiological functions and good quality of life. We should not indefinitely expand the scope of resection for the blind pursuit of thoroughness for a radical cure, as this will reduce the patient’s quality of life, and we should not pursue a good quality of life at the expense of the principle of a radical cure. The choice of the surgical approach for GCA has a significant impact on the surgical treatment effect, and a reasonable choice should be made based on the patient’s overall condition before surgery. Because the transthoracic approach is better than the transabdominal approach in terms of the thoroughness of the operation and better than the thoracoabdominal approach in terms of degree of trauma, the transthoracic approach should be the first choice if the patient’s general condition permits. Based on the large-scale data and long-term follow-up of this study, the following suggestions are made for the selection of the GCA surgical approach: (a) the transthoracic approach may be preferred; (b) if it is estimated that the tumor cannot be resected before the operation, transabdominal exploration can be performed; if necessary, a thoracoabdominal approach may be used; and (c) for elderly patients with cardiopulmonary insufficiency, a transabdominal approach can be considered.

The decision of which surgical approach to use depends on the preference and experience of the operating surgeon and the patient’s baseline physiological characteristics, such as complications and level and stage of the tumor. In the present study, there was no randomization, and the patients were selected on the basis of the surgeon’s criteria, which may introduce selection bias. In addition, we did not evaluate complications and the use of postoperative chemoradiotherapy, which may interfere with the results observed. Therefore, while the results of this study have certain implications on some issues, the results still need to be cautiously interpreted. A large study with randomization is required to obtain a stronger level of evidence.

## Conclusions

Thoracoabdominal approach and transabdominal approach were shown to be poor prognostic factors. Patients with (locally advanced) GCA may benefit from the transthoracic approach. Furthermore, when the tumor was in the early or advanced stage, the choice of surgical approach had little effect on the survival of patients with GCA; when the tumor was in the locally advanced stage, the surgical approach had an impact on the survival rate, and the transthoracic approach could be selected since it may lead to a higher survival rate. Further prospective randomized clinical trials are necessary.

## Supplementary Information

**Additional file 1:** **Supplemental Table S1.** Comparison of lymph node variables among the three surgical approaches for 4644 GCA patients. **Supplemental Figure S1.** Kaplan–Meier curves comparing different surgical approaches of GCA patients in different tumor stages: (A) Stage 0 - I, (B) Stage II, (C) Stage III, and (D) Stage IV. **Supplemental Figure S2.** Kaplan–Meier curves comparing different surgical approaches of GCA patients in N0 stage (A) and N1 stage (B). **Supplemental Figure S3.** Kaplan–Meier curves comparing surgical approach of GCA patients in different periods: 1974–1999 (A); 2000–2011 (B); and 2012–2020 (C).

## Data Availability

The datasets used and/or analyzed during the current study are available from the corresponding author on reasonable request.
