# Supplementary material for: Transthoracic, thoracoabdominal, and transabdominal surgical approaches for gastric cardia adenocarcinomas: a survival evaluation based on a cohort of 7103 patients
Source: World J Surg Oncol. 2022 Jun 28;20:217. doi: 10.1186/s12957-022-02680-5 (PMC9238161; doi:10.1186/s12957-022-02680-5)
Supplement: Supplementary file 1 — Additional file 1: Supplemental Table S1. Comparison of lymph node variables among the three surgical approaches for 4644 GCA patients. Supplemental Figure S1. Kaplan–Meier curves comparing different surgical approaches of GCA patients in different tumor stages: (A) Stage 0 - I, (B) Stage II, (C) Stage III, and (D) Stage IV. Supplemental Figure S2. Kaplan–Meier curves comparing different surgical approaches of GCA patients in N0 stage (A) and N1 stage (B). Supplemental Figure S3. Kaplan–Meier curves comparing surgical approach of GCA patients in different periods: 1974–1999 (A); 2000–2011 (B); and 2012–2020 (C). [file 12957_2022_2680_MOESM1_ESM.zip › FigureS1_ESM.pdf]

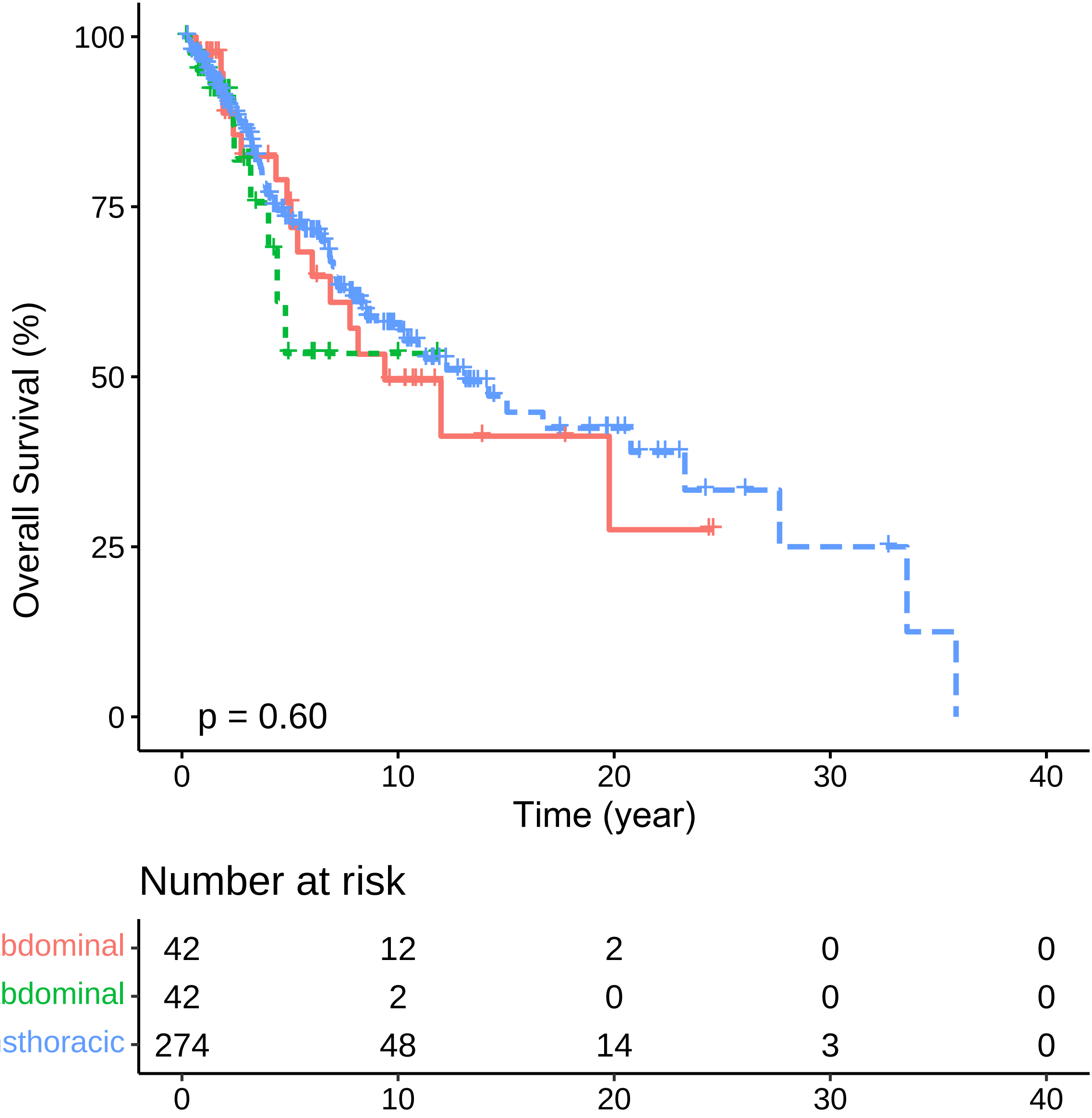

Overall Survival (%)
100
75
50
25
0
p = 0.60
0
10
20
30
40
Time (year)
Number at risk
Thoracoabdominal
Transabdominal
Transthoracic
42 12 2 0 0
42 2 0 0 0
274 48 14 3 0
0
10
20
30
40

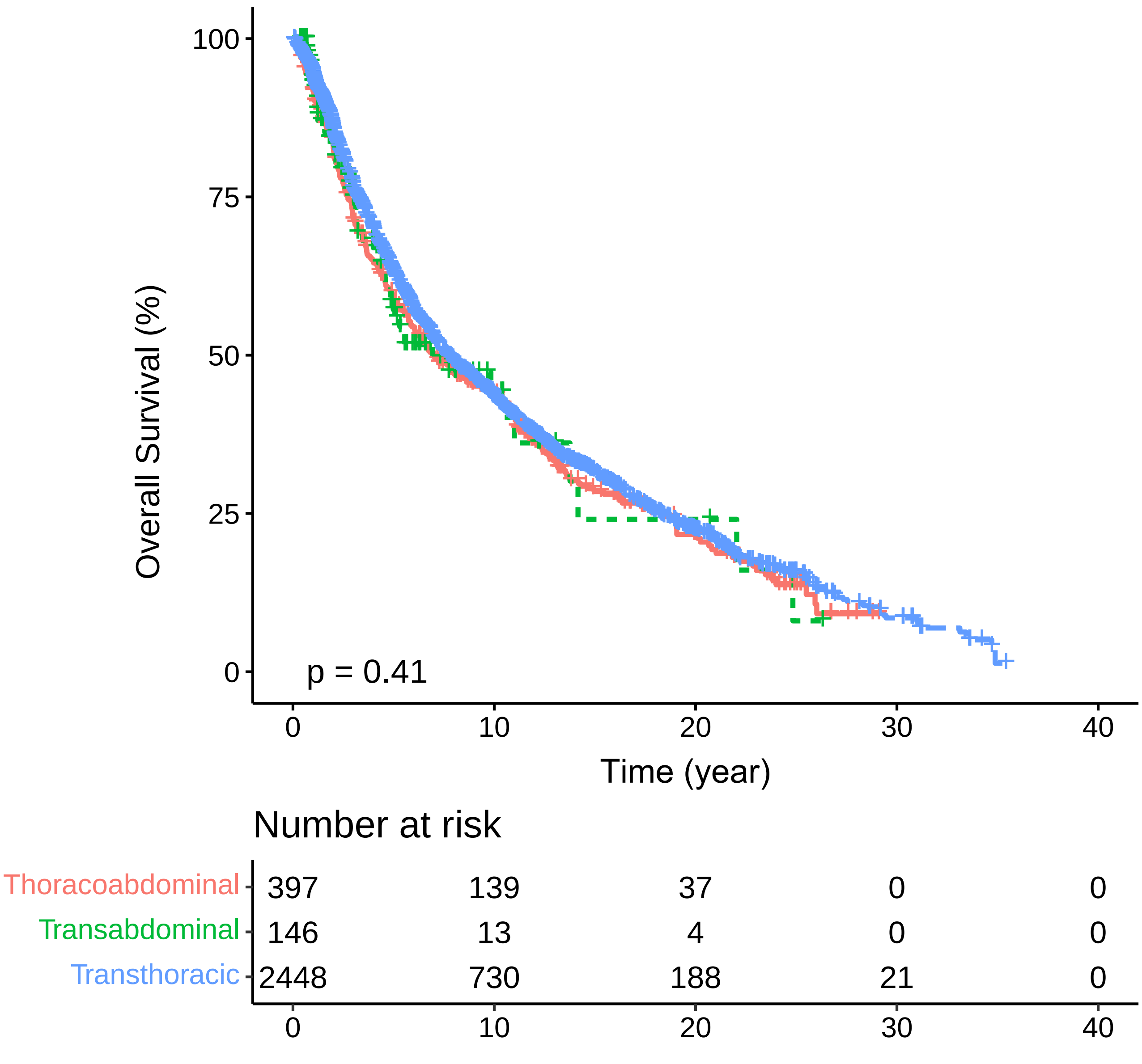

Overall Survival (%)
100
75
50
25
0
p = 0.41
0
10
20
30
40
Time (year)
Number at risk
Thoracoabdominal 397 139 37 0 0
Transabdominal 146 13 4 0 0
Transthoracic 2448 730 188 21 0
0
10
20
30
40

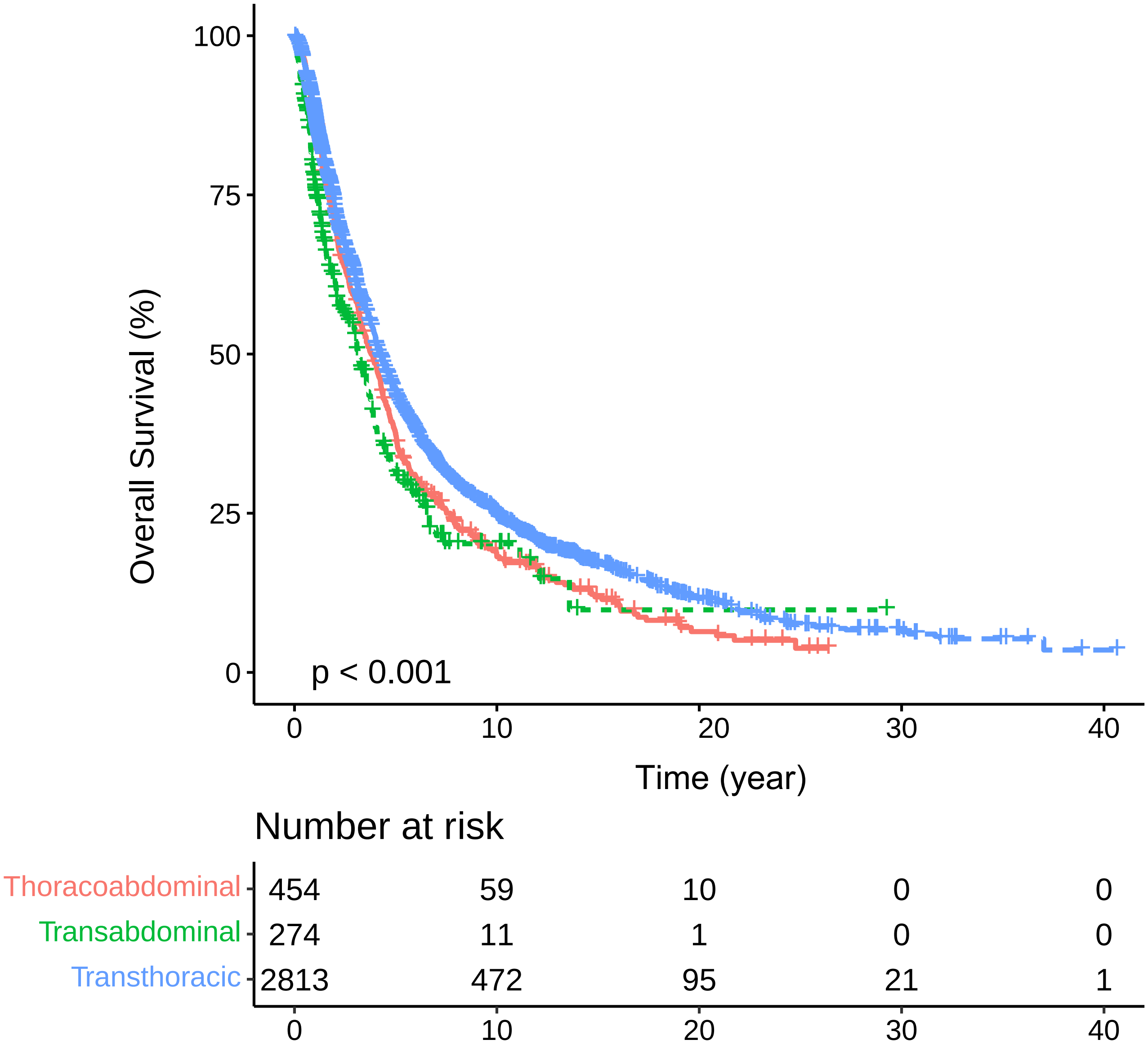

Overall Survival (%)
100
75
50
25
0
p < 0.001
0
10
20
30
40
Time (year)
Number at risk
Thoracoabdominal 454 59 10 0 0
Transabdominal 274 11 1 0 0
Transthoracic 2813 472 95 21 1
0
10
20
30
40

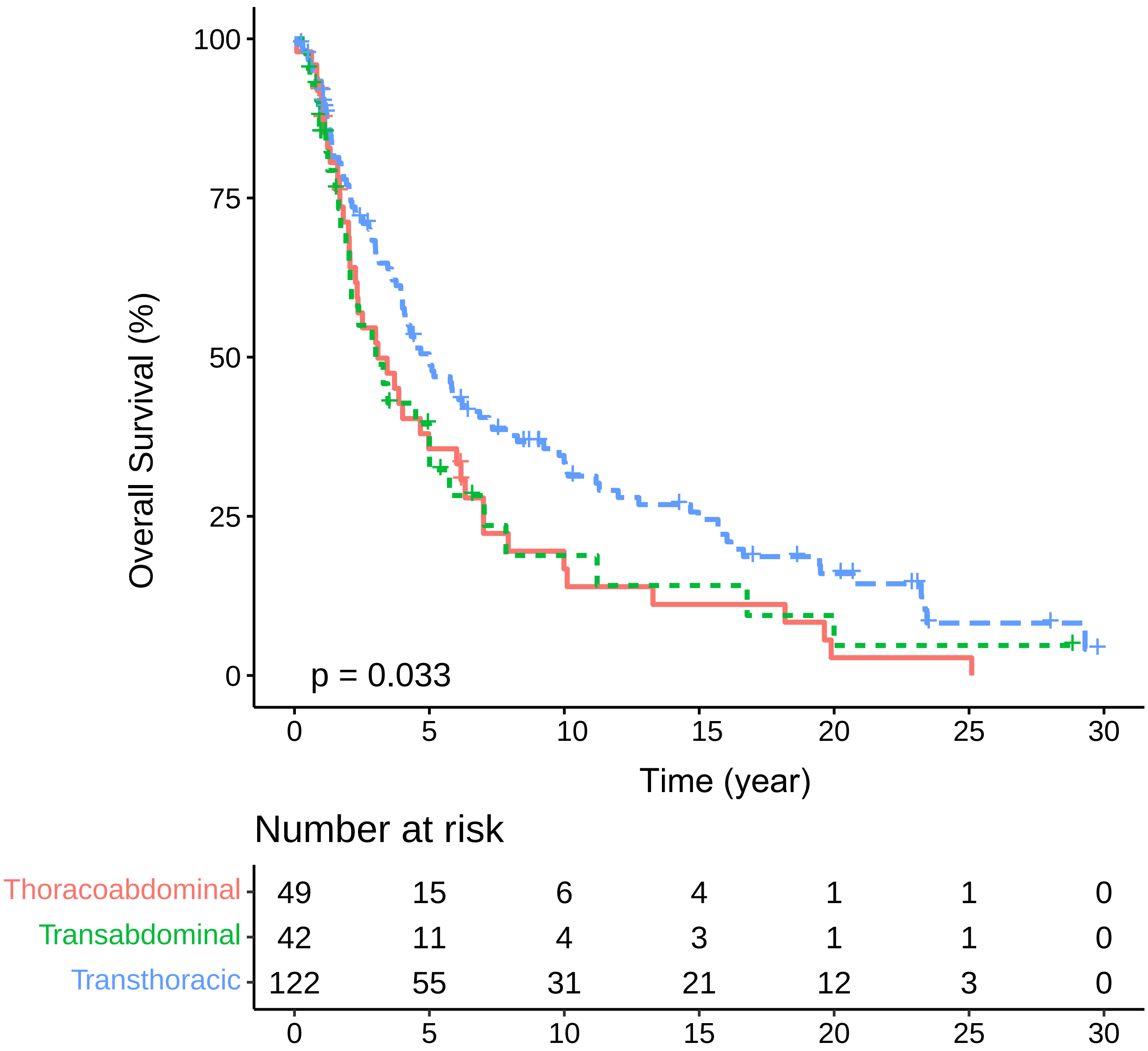

Overall Survival (%)
100
75
50
25
0
p = 0.033
0
5
10
15
20
25
30
Time (year)
Number at risk
Thoracoabdominal 49 15 6 4 1 1 0
Transabdominal 42 11 4 3 1 1 0
Transthoracic 122 55 31 21 12 3 0
0
5
10
15
20
25
30
