# Supplementary material for: Transthoracic, thoracoabdominal, and transabdominal surgical approaches for gastric cardia adenocarcinomas: a survival evaluation based on a cohort of 7103 patients
Source: World J Surg Oncol. 2022 Jun 28;20:217. doi: 10.1186/s12957-022-02680-5 (PMC9238161; doi:10.1186/s12957-022-02680-5)
Supplement: Supplementary file 1 — Additional file 1: Supplemental Table S1. Comparison of lymph node variables among the three surgical approaches for 4644 GCA patients. Supplemental Figure S1. Kaplan–Meier curves comparing different surgical approaches of GCA patients in different tumor stages: (A) Stage 0 - I, (B) Stage II, (C) Stage III, and (D) Stage IV. Supplemental Figure S2. Kaplan–Meier curves comparing different surgical approaches of GCA patients in N0 stage (A) and N1 stage (B). Supplemental Figure S3. Kaplan–Meier curves comparing surgical approach of GCA patients in different periods: 1974–1999 (A); 2000–2011 (B); and 2012–2020 (C). [file 12957_2022_2680_MOESM1_ESM.zip › FigureS3_ESM.pdf]

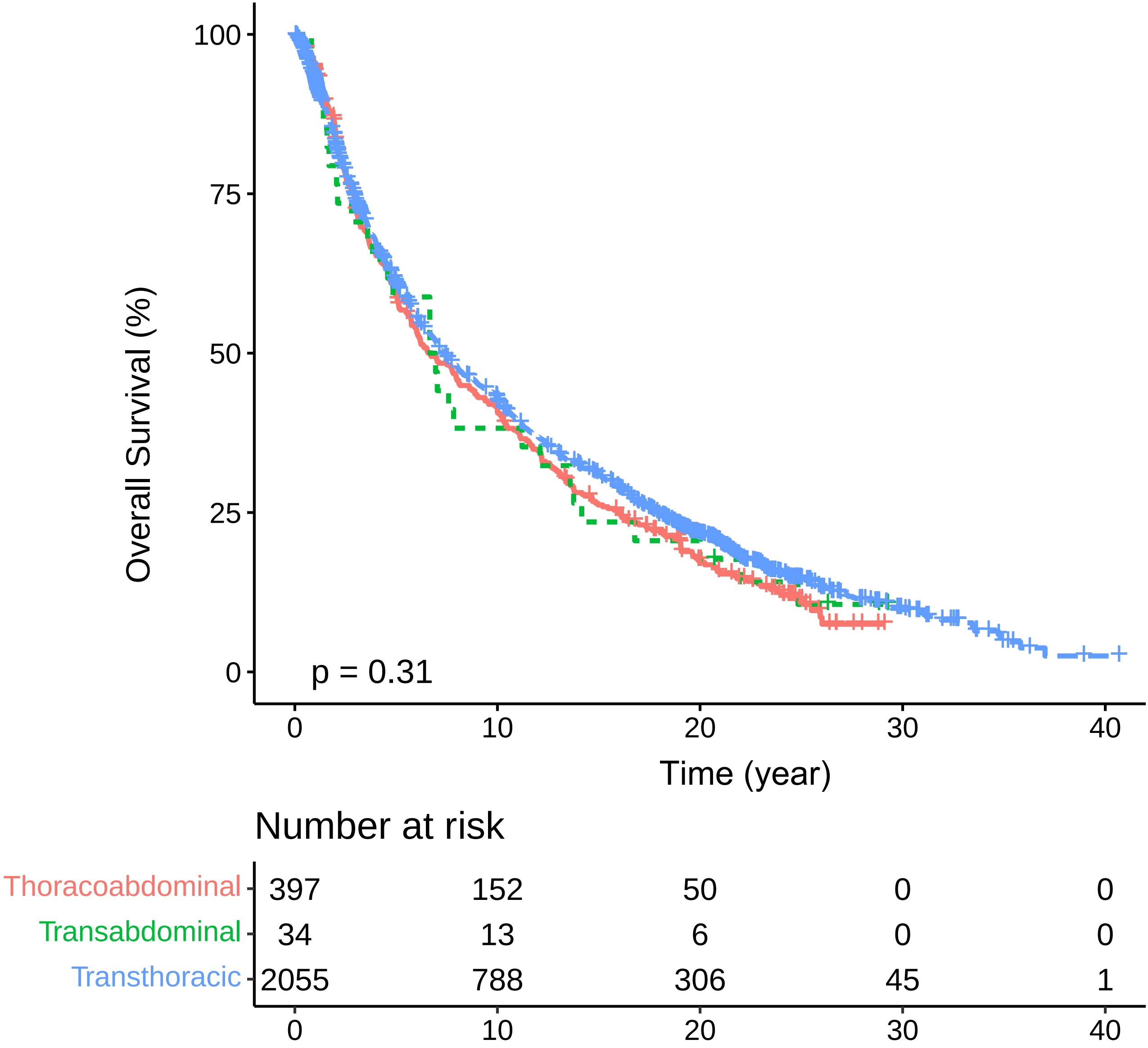

Overall Survival (%)
100
75
50
25
0
p = 0.31
0
10
20
30
40
Time (year)
Number at risk
Thoracoabdominal
Transabdominal
Transthoracic
397
152
50
0
0
34
13
6
0
0
2055
788
306
45
1
0
10
20
30
40

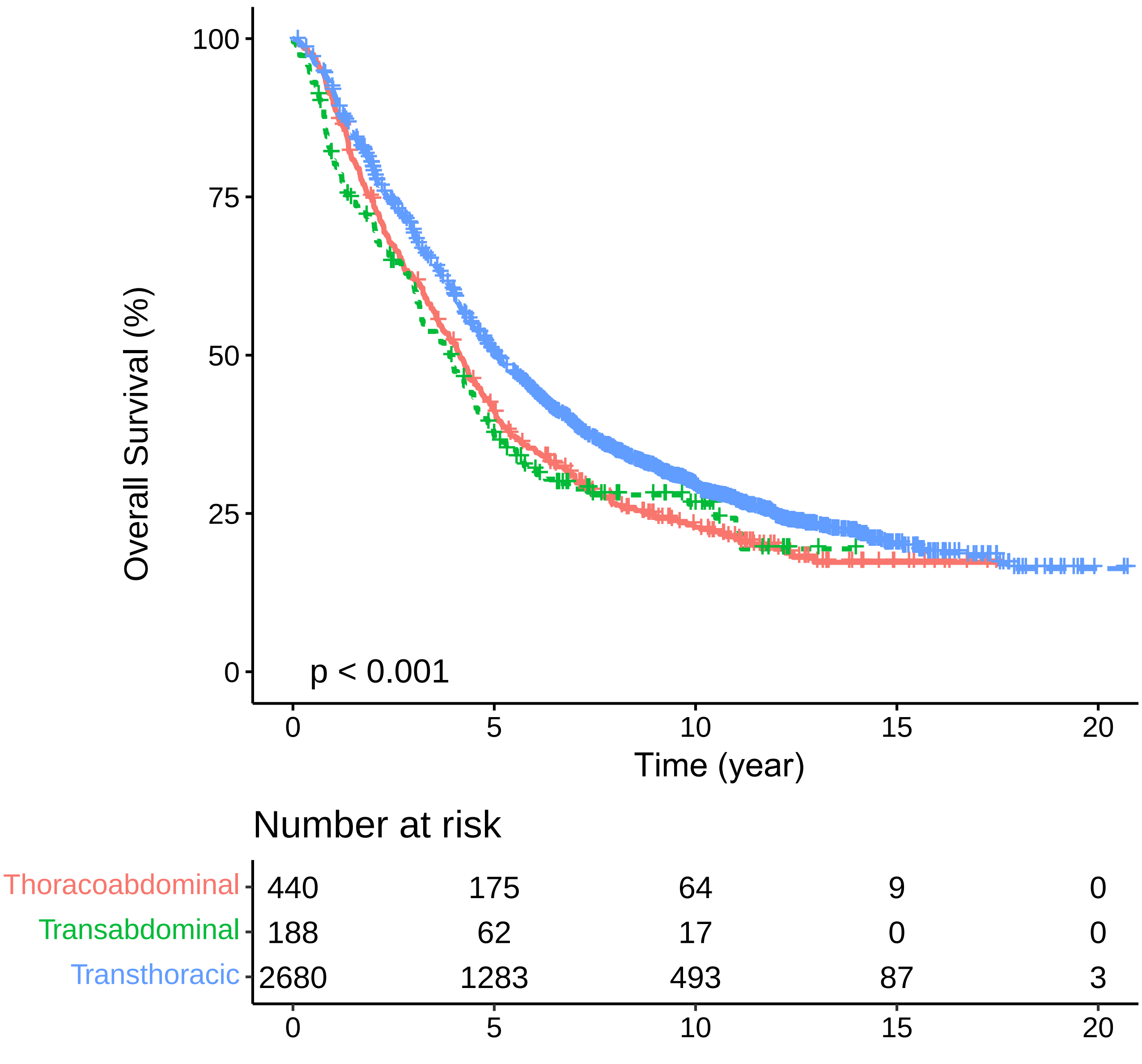

Overall Survival (%)
100
75
50
25
0
p < 0.001
0
5
10
15
20
Time (year)
Number at risk
Thoracoabdominal 440 175 64 9 0
Transabdominal 188 62 17 0 0
Transthoracic 2680 1283 493 87 3
0
5
10
15
20

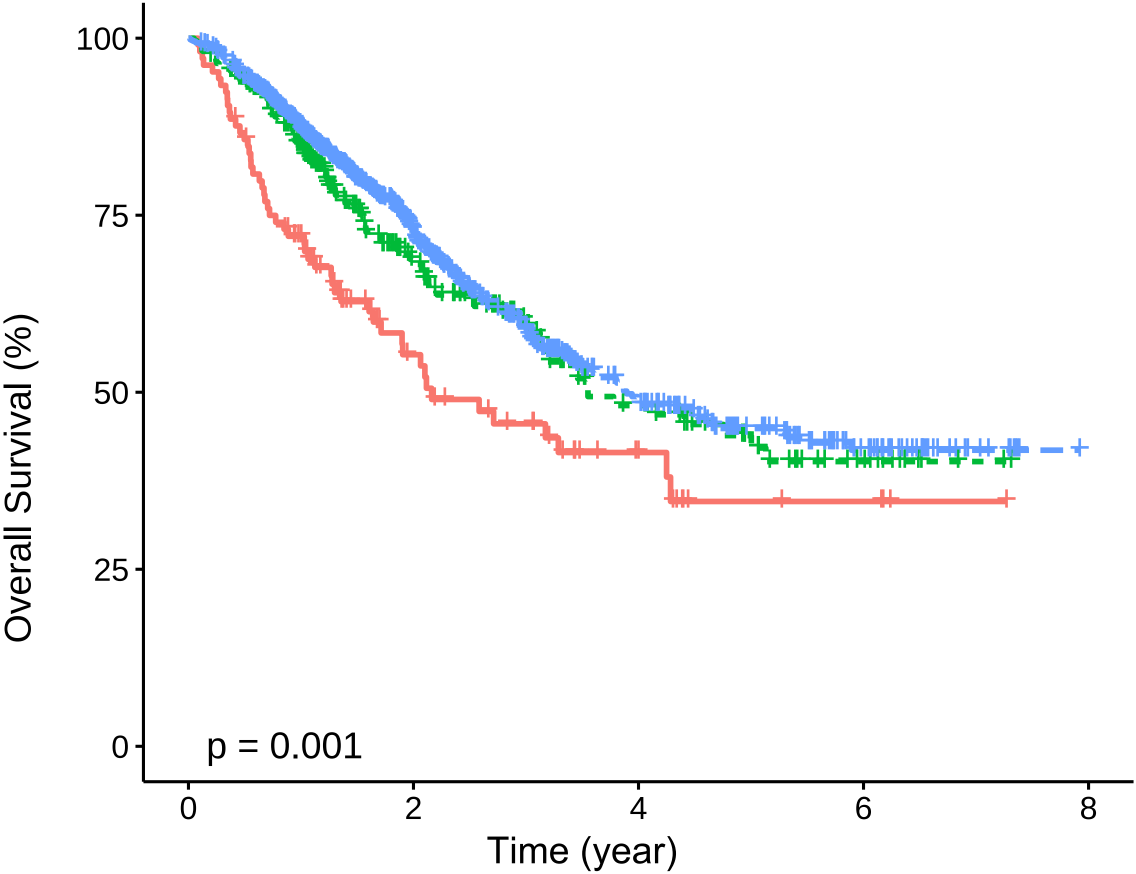


Number at risk

| | 0 | 2 | 4 | 6 | 8 |
|---|---|---|---|---|---|
| Thoracoabdominal | 105 | 35 | 13 | 4 | 0 |
| Transabdominal | 282 | 98 | 37 | 12 | 0 |
| Transthoracic | 922 | 376 | 127 | 38 | 0 |
